# Successful treatment of a huge hepatic carcinoma with right portal vein thrombosis: A case report

**DOI:** 10.1097/MD.0000000000019636

**Published:** 2020-04-24

**Authors:** Kezhong Tang, Bo Zhang, Linping Dong, Lantian Wang, Yecheng Jin, Zhe Tang

**Affiliations:** a Department of Surgery, 2^nd^ Affiliated Hospital; b Department of Pharmacy, Affiliated Sir RunRun Shaw Hospital, Zhejiang University School of Medicine, Hangzhou, PR China.

**Keywords:** associating liver partition and portal vein ligation for staged hepatectomy, portal vein thrombosis, radiofrequency ablation

## Abstract

**Introduction:**

Unlike the traditional associating liver partition and portal vein ligation for staged hepatectomy, it is still controversial whether patients with portal vein thrombosis can receive benefits from liver partition.

**Patient concerns:**

Right upper abdominal distension for 2 months.

**Diagnosis:**

Hepatocellular carcinoma with portal vein invasion

**Intervention:**

Radiofrequency-assisted liver partition with portal vein ligation (RALPP)

**Outcomes:**

Disease-free survival: 3 months, overall survival: 7 months

**Conclusion:**

Our results advocate this variation of RALPP for use in patients with huge HCC with portal vein invasion, without enough future liver remnant. Patients can receive benefits from the operation, including a shorter operation time, better recovery, and lower overall costs of the 2-stage procedure.

## 1 Introduction

Over the years, various methods have been used clinically to induce hypertrophy of the future liver remnant (FLR) preoperatively, with the aim of reducing postoperative complications and increasing the proportion of patients suitable for resection. Portal vein embolization (PVE) is the standard technique used clinically in patients with insufficient FLR before liver resection, and it can result in an increase in the FLR volume by 11.9%.^[1]^ Schnitzbauer et al^[2]^ proposed the alternative surgical method of associating liver partition and portal vein ligation for staged hepatectomy (ALPPS), where a much greater increase in the FLR volume of 74% was seen within a much shorter time. However, the morbidity from this procedure^[2–5]^ is 33% to 64% compared with 16% after PVE.^[1]^ The morbidity after ALPPS is increased because of a high rate of postoperative bile leaks. Many innovations for ALPPS have been developed to decrease morbidity and mortality while maintaining a more rapid and robust FLR hypertrophy.^[6–9]^

The use of laparoscopy during both stages of ALPPS has been performed successfully.^[10,11]^ On the basis of the small numbers available, there are subjective reports of decreased adhesions encountered after a laparoscopic first stage.^[10,12]^ However, a laparoscopic approach is believed to be likely associated with an increase in technical difficulty.^[12]^ Radiofrequency ablation (RFA) is an established technique for the treatment of hepatic tumors that uses rapidly alternating currents to produce coagulative necrosis of the hepatic parenchyma.^[13]^ Gall et al^[8]^ reported a modification of ALPPS by using RFA during a laparoscopic first stage to produce a line of avascular necrosis along the future line of transection, termed radiofrequency-assisted liver partition with portal vein ligation (RALPP). Hypothetically, this hemiliver technique induces FLR hypertrophy without a physical parenchymal split. The 4 patients treated laparoscopically in the study experienced no serious complications (Clavien-Dindo grade ≥IIIb).

According to the American Association for the Study of the Liver Disease/Barcelona Clinic for Liver Cancer Staging System and treatment guidelines, portal vein tumor thrombosis (PVTT) is regarded as an advanced stage of the disease with almost no hope for a cure.^[14]^ The only proposed treatment option for this group of patients is sorafenib chemotherapy, and the reported median survival time of patients with advanced hepatocellular carcinoma (HCC) treated with sorafenib is as short as 10.7 months.^[15]^ Aggressive surgical resection for HCC with vascular invasion has been proposed by several tertiary centers.^[16–18]^ Here, we describe a case of a huge hepatic carcinoma with right portal vein thrombosis that was successfully treated by RALPP using a laparoscopic approach, without portal vein ligation.

## 2 Case report

The patient provided informed consent for publication of this case.

**Figure 1:**
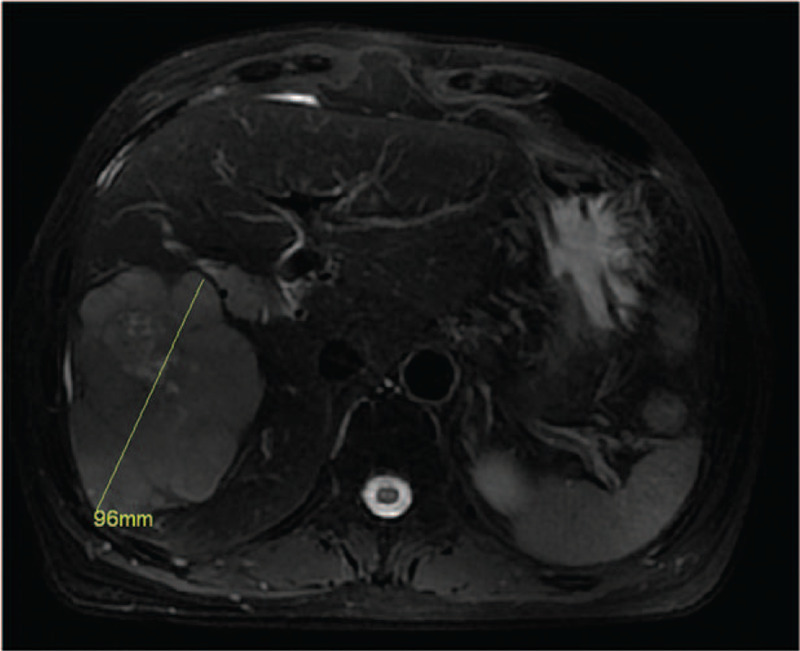
Magnetic resonance imaging scan shows a right huge hepatic mass with portal vein invasion.

**Figure 2:**
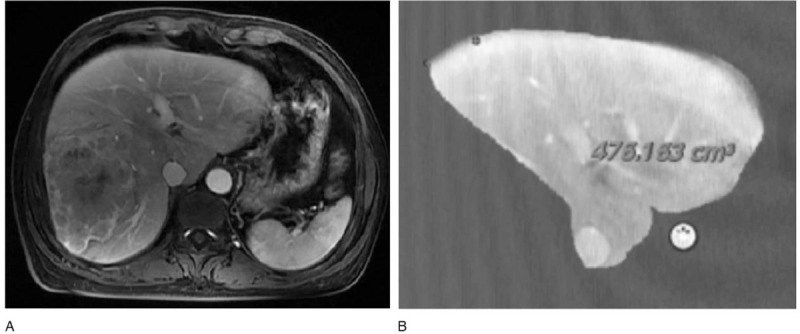
Magnetic resonance imaging scan shows (A) a huge mass in the right liver and (B) the future liver remnant after right hepatectomy.

A 42-year-old woman presented with a history of chronic hepatitis B virus (HBV) and was being evaluated for antiviral treatment. As part of her work-up, ultrasonography of the liver was performed and showed an incidental liver mass. A confirmatory magnetic resonance imaging (MRI) showed a huge mass with several metastases surrounding the right liver with right portal vein invasion that measured 9.6 × 9.1 cm (Fig. 1). The tumor characteristics were combined with a markedly elevated alpha-fetoprotein level (>20,000 ng/mL) and no evidence of splenomegaly or portal hypertension, which were consistent with HCC. Because of the presence of tumor thrombus, the patient was not a liver transplant candidate. MRI volumetrics of the liver showed that if the required right hepatectomy was done, the FLR (segments 1, 2, and 3), which constituted 19% of the total liver volume, would likely be insufficient for postoperative recovery (Fig. 2A and B). As a result of complete right portal vein tumor thrombosis (type II portal vein tumor), right PVE or portal vein ligation would not be necessary.

**Figure 3:**
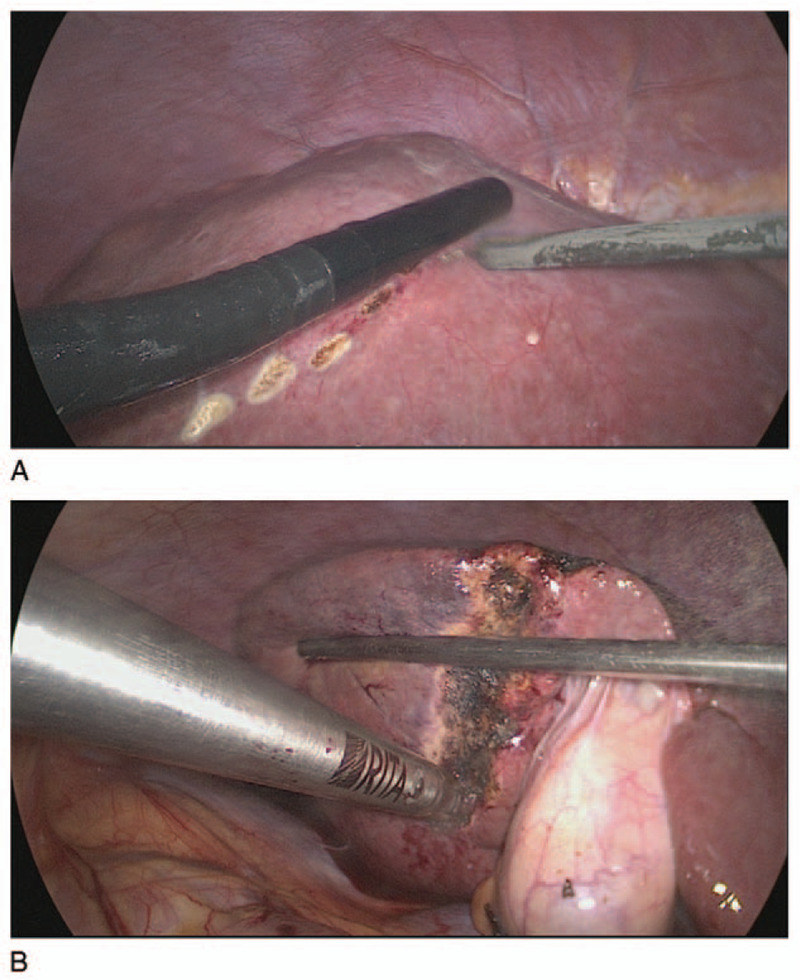
(A) A line for liver partition is made on the surface of the liver under ultrasound guidance. (B) Avascular necrosis along the future line of transection is produced by using the Habib Sealer (LH4X, Rita).

Plans were made to use RALPP without portal vein ligation (RALP) with the laparoscopic approach to induce rapid hypertrophy of the FLR. After appropriate preoperative preparation, the patient was taken to the operating room for stage I of RALP. After the induction of general anesthesia, the patient was placed in the supine position. Pneumoperitoneum (carbon dioxide at 12 mm Hg) was established, and the abdomen was explored with a 30° laparoscope through a 10-mm umbilical port. Another 10-mm subxiphoid port was created at the midline of the abdomen. One additional 10-mm right or left lateral subcostal port was placed. Laparoscopic cholecystectomy was performed beforehand. Under ultrasonographic guidance, a line for the liver partition on the surface of liver was created by using a laparoscopic electric knife. The Habib Sealer (LH4X, Rita) was introduced into the peritoneal cavity through the subxiphoid port with laparoscopic visualization and punctured the line created above (Fig. 3A and B). The radiofrequency ablation process was monitored by intraoperative ultrasonography. The FLR (segments 1, 2, and 3) was evaluated using laparoscopic Doppler ultrasonography, which showed good arterial and portal inflow. A laparoscopic biopsy of the liver tumor was not performed before the ablation to avoid unnecessary bleeding. The laparoscopic operation was finished in the standard manner, and the patient was taken to the ward.

**Figure 4:**
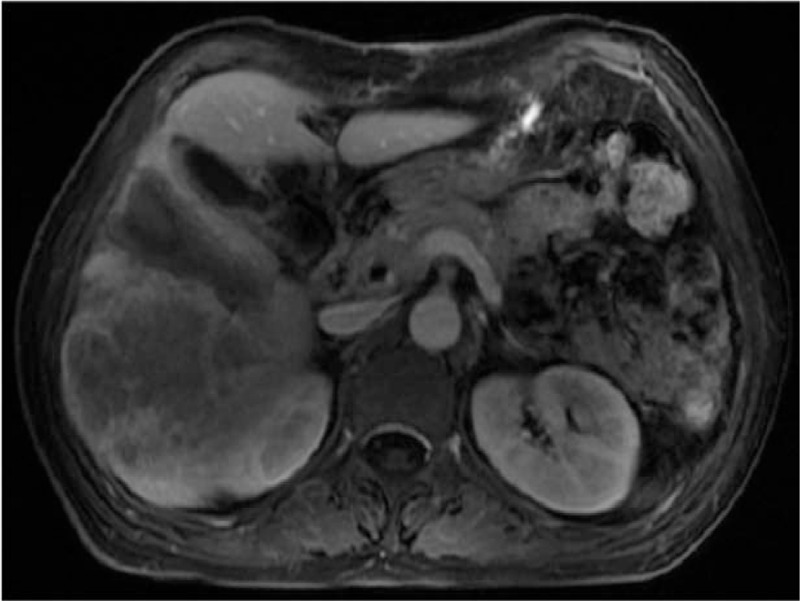
Magnetic resonance imaging scan shows coagulative necrosis of the hepatic parenchyma created by radiofrequency ablation.

On postoperative day (POD) 3, postoperative MRI confirmed coagulative necrosis of the hepatic parenchyma created by RFA to cease blood flow from the FLR to the diseased hemiliver (Fig. 4). Aspartate aminotransferase (AST) and alanine aminotransferase (ALT) levels were primarily significantly elevated from 111 U/L and 49 U/L to 391 U/L and 168 U/L on the first POD, respectively, but they consistently decreased to 115 U/L and 51 U/L on POD 5. The patient returned home on POD 7, and stage II of RALP was planned for 2 weeks after stage I of RALP to induce hypertrophy of the FLR.

**Figure 5:**
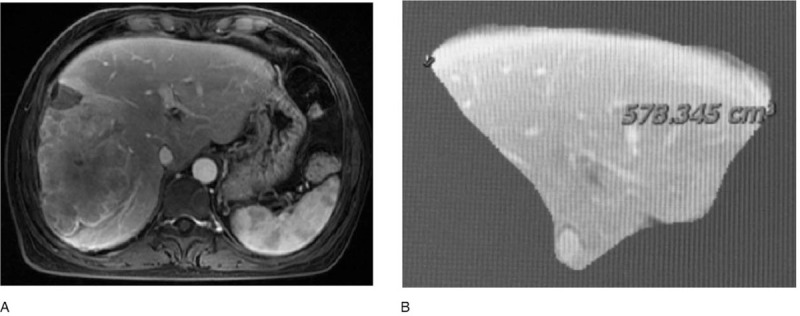
Magnetic resonance imaging scan shows (A) a huge mass in the right liver and (B) the future liver remnant after right hepatectomy.

Further MRI volumetry conducted on POD 15 showed an FLR volume of 578 cm^3^ (approximately 35.2% of the total liver volume; FLR/body weight ratio of 0.52), resulting in a volume increase of about 21.5% following stage I of RALP (Fig. 5A and B). Total bilirubin, AST, and ALT levels returned to 11.2 μmol/L, 53 U/L, and 21 U/L, respectively. The patient was taken back to the operating room on POD 25 and underwent stage II of RALP.

**Figure 6:**
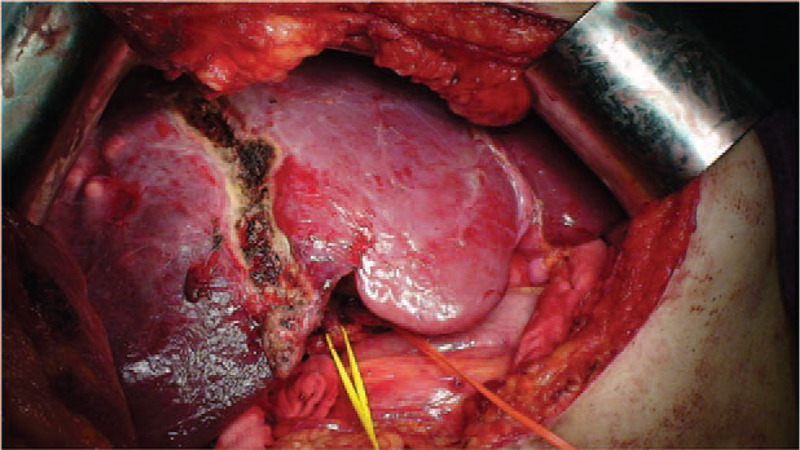
The line of avascular necrosis is created in stage I of radiofrequency ablation assisted-associating liver partition and portal vein ligation for staged hepatectomy without portal vein ligation and produced by radiofrequency ablation.

The right liver, which was full of the tumor, was split along the line of avascular necrosis created in stage I of RALP produced by RFA (Fig. 6). Intraoperative Doppler ultrasonography of the FLR was performed again and showed good arterial and portal venous inflow as well as excellent hepatic venous outflow. The FLR was secured to the anterior abdominal wall at the falciform ligament, a surgical drain was left at the resection margin, and the abdominal wall was closed in the standard manner. Then, the patient was transported to the ward and left the hospital on POD 11.

The follow-up period after the operation lasted for 7 months. After 3 months of remission, HCC relapsed near the edge of resection. The patient died at 7 months postoperatively.

## 3 Discussion

Treatment of large advanced HCCs is technically challenging. Evidence has shown that hepatectomy still provides a better survival outcome than nonsurgical approaches.^[19]^ The decision to perform hepatectomy or nonsurgical approaches usually depends on the balance between surgical safety and anticipated survival performance. This patient was not eligible for liver transplantation because her tumor was too large and had invaded the right portal vein. Without appropriate hypertrophy, extended right hepatectomy would be risky because her liver remnant would be too small. She was also not a good candidate for right PVE for inducing hypertrophy of the FLR because she already had auto-embolization of the right portal vein branches. In the treatment of large HCCs, the role of PVE is restricted. Intrahepatic metastasis can occur within a short period.

ALPPS appears to be an ideal solution for patients with HCC who would have a small liver remnant after hepatectomy. Since the original description by Schnitzbauer et al,^[2]^ the ALPPS technique has undergone many modifications, sparking both intense enthusiasm and skepticism alike among the surgical community. ALPPS is superior to PVE in terms of FLR, but it is associated with greater morbidity and mortality, particularly due to bile leaks and hemorrhage after the initial procedure.^[4,5,20]^ Since its introduction, ALPPS now serves as an umbrella term under which many variations and adaptations exist. A main driver for further innovation is the goal of decreasing morbidity and mortality while maintaining a more rapid and robust FLR hypertrophy.

RFA is an established technique for the treatment of hepatic tumors that uses rapidly alternating currents to produce coagulative necrosis of the hepatic parenchyma. There is little research about using RFA in the first stage of ALPPS.^[8]^ To our knowledge, this is the first report of using the Habib Sealer in ALPPS with the laparoscopic approach without portal vein ligation. We think our success in the present case was a result of the following maneuvers.

(1) We did not conduct right portal vein ligation because the right PVTT had already stopped portal vein inflow. Aggressive treatment of the PVTT might have destroyed the stability of the thrombosis and cause intrahepatic or extrahepatic metastasis. Therefore, in this case, we chose to maintain stability of the thrombosis, which also simplified stage I of RALP. The length of stage I of RALP was as short as 30 minutes with the laparoscopic approach. The patient recovered and returned home on POD 7.
(2) Bile leaks are a clinically important source of complications associated with ALPPS and can lead to serious morbidity secondary to sepsis.^[21]^ In our patient, transection was performed through an avascular groove created by the Habib Sealer in the second stage without the need for containment of any transection surface between the stages. We did not perform ligation of the bile duct during the first stage, and this may be beneficial in decreasing biliary fistula and leakage from the cut surface.
(3) Laparoscopic surgery is associated with a decrease in adhesions, operative trauma, and postoperative duration of stay.^[22]^ By taking advantage of the Habib Sealer to split the liver, there was little adhesion during stage II of RALP in our case. The patient was able to leave hospital before the second stage, thereby decreasing overall costs of the 2-stage procedure.

The mechanism behind the increased FLR hypertrophy in ALPPS is yet to be fully determined. Traditionally, this hypertrophy was believed to be related to the cessation of blood flow between the diseased segments and the FLR. For all variations of ALPPS, FLR hypertrophy ranged between 60%^[6]^ and 90%.^[11]^ In our case, there was only a 21.5% increase of FLR from 476 to 578 mL. The main reason for the low growth of the FLR may be the right PVTT before stage I of RALP. With the traditional evaluation of FLR hypertrophy, there are 2 reasons for low growth of the FLR: splitting the liver and ligation of the portal vein. In this case, the right portal vein had been invaded by the tumor, so the left liver might have enlarged to 476 mL preoperatively. The 21.5% growth of the FLR in this case was due to splitting of the liver by the Habib Sealer.

To our knowledge, this is the first reported case of using the variation of RALPP with a laparoscopic approach without portal vein ligation in a patient with chronic HBV and portal vein thrombosis. Our results advocate the use of this variation of RALPP in patients with a huge HCC with portal vein invasion, without sufficient FLR. Patients can receive benefits from the operation, including a shorter operative time, better recovery, and lower overall costs of the 2-stage procedure.

## 4 Conclusion

RALP allowed for rapid hypertrophy of the FLR without postoperative liver failure. This technique can be performed easily with the laparoscopic approach, thus potentially increasing the number of patients who would be candidates for curative resection. Further studies will be important to define optimal use of this technique and increased FLR hypertrophy.

## Author contributions

**Conceptualization:** Kezhong Tang.

**Data curation:** Kezhong Tang.

**Formal analysis:** Kezhong Tang.

**Funding acquisition:** Kezhong Tang.

**Methodology:** Linping Dong.

**Project administration:** Linping Dong, Zhe Tang.

**Resources:** Zhe Tang.

**Software:** Zhe Tang.

**Supervision:** Lantian Wang, Zhe Tang.

**Validation:** Bo Zhang, Linping Dong, Lantian Wang.

**Visualization:** Bo Zhang, Lantian Wang.
